# Theoretical considerations and supporting evidence for the primary role of source geometry on field potential amplitude and spatial extent

**DOI:** 10.3389/fncel.2023.1129097

**Published:** 2023-03-30

**Authors:** Oscar Herreras, Daniel Torres, Valeriy A. Makarov, Julia Makarova

**Affiliations:** ^1^Laboratory of Experimental and Computational Neurophysiology, Department of Translational Neuroscience, Cajal Institute, Spanish National Research Council, Madrid, Spain; ^2^Institute for Interdisciplinary Mathematics, School of Mathematics, Universidad Complutense de Madrid, Madrid, Spain

**Keywords:** field potential, source geometry, spatial reach, volume conduction, network oscillations, current source, LFP, source localization

## Abstract

Field potential (FP) recording is an accessible means to capture the shifts in the activity of neuron populations. However, the spatial and composite nature of these signals has largely been ignored, at least until it became technically possible to separate activities from co-activated sources in different structures or those that overlap in a volume. The pathway-specificity of mesoscopic sources has provided an anatomical reference that facilitates transcending from theoretical analysis to the exploration of real brain structures. We review computational and experimental findings that indicate how prioritizing the spatial geometry and density of sources, as opposed to the distance to the recording site, better defines the amplitudes and spatial reach of FPs. The role of geometry is enhanced by considering that zones of the active populations that act as sources or sinks of current may arrange differently with respect to each other, and have different geometry and densities. Thus, observations that seem counterintuitive in the scheme of distance-based logic alone can now be explained. For example, geometric factors explain why some structures produce FPs and others do not, why different FP motifs generated in the same structure extend far while others remain local, why factors like the size of an active population or the strong synchronicity of its neurons may fail to affect FPs, or why the rate of FP decay varies in different directions. These considerations are exemplified in large structures like the cortex and hippocampus, in which the role of geometrical elements and regional activation in shaping well-known FP oscillations generally go unnoticed. Discovering the geometry of the sources in play will decrease the risk of population or pathway misassignments based solely on the FP amplitude or temporal pattern.

## 1. Space, finally

Field potentials (FPs) reflect the operation of neuronal networks and as such, they are widely used to explore brain physiology and pathology. Amongst their advantages, FP waves can be recorded with high resolution, which makes them optimal signals to deal with the transient activation of neuronal populations. Moreover, their population nature provides an opportunity to monitor functional groups of neurons or cell assemblies (Fernández-Ruiz et al., 2012), these being viewed as the processing units of high-order structures (Abeles, 1991; Sakurai, 1998; Hanson et al., 2012; Papadimitriou et al., 2020). Given the primarily synaptic nature of FPs (Elul, 1971; Haberly and Shepherd, 1973), this paves the way to identify the anatomical correlates of any given activity.

Identifying the anatomical sources of brain potentials is not straightforward. We are used to visualizing FPs as pen traces that represent voltage fluctuations over time. However, brain potentials are space-varying three-dimensional signals that represent a mix of multiple sources (Supplementary Video 1), which has made their analysis difficult due to intrinsic restrictions and technical constraints. The poor access to the complex time-varying geometry of current sources has disengaged FP management from its causes, as manifested by the limited understanding of the factors that determine their amplitude and spatial extent. Decades of single-electrode recordings have led researchers to consider voltages at specific sites rather than fields in space, forging a widespread and erroneous view of the FPs generated by local neurons, even yielding the term local field potential (LFP) despite the fact that this does not make much sense in terms of physics. Indeed, electric fields extend beyond the populations that inject the current into the extracellular space and they may reach distant sensors while retaining significant amplitude (Lorente de Nó, 1947; Woodbury, 1960; Nunez and Srinivasan, 2006; Hales and Pockett, 2014). In the brain, such fields are commonly referred to as volume conducted potentials and they are often deemed negligible on the erroneous assumption that they decay dramatically as the distance from the neuronal source augments. Such an expectation is derived from the theoretical decay of the potentials from an ideal dipole (1/distance cubed). However, the complex filamentous structure of neurons prevents them from behaving as ideal dipoles, since the domains with net inward and outward currents (extracellular sinks and sources) have highly unequal spatial distribution and current density. In practice, each patch of membrane that experiences a current flow counts toward establishing extracellular potentials. Consequently, during population activation, the composite FP depends on the instantaneous value of current density and position of all active neural elements in 3D space, which leads to non-intuitive spatial distributions. Indeed, the activation of brain populations may or may not produce FPs (Lorente de Nó, 1947). As such, FPs may be negligible even at the center of the active population, or the amplitude may be even larger out of it. Both circumstances are incompatible with strict relationships with distance (Fernández-Ruiz et al., 2013). Evidence shows that FPs recorded in many brain structures get there by passive spread in the volume and actually arise in other regions (Carmichael et al., 2017; Lalla et al., 2017; Parabucki and Lampl, 2017; Bertone-Cueto et al., 2020). Meanwhile regions that produce significant FPs as the cortex and hippocampus also extend their potentials into each other’s domains (Torres et al., 2019), making FP waveforms multisource and site-dependent (Herreras et al., 2022). Significantly, electroencephalograms (EEGs) themselves are an entirely volume-conducted conglomerate of FPs (Nunez and Srinivasan, 2006).

The extension of FPs in space is generally poorly addressed in the literature. Some authors propose a generic spatial reach for any FPs, whereas others point to specific factors as the magnitude and size of the current source as the main determinants (Berens et al., 2008; Katzner et al., 2009; Liu et al., 2015). However, when referring to brain sources neither is correct, as it will become evident by the data reviewed below. The improvements in experimental capabilities in the last decade have finally allowed spatial information to be drawn from FP sources (e.g., multisite recordings: Benito et al., 2014; Herreras et al., 2015; Whitmore and Lin, 2016), which is rapidly changing the erroneous idea that they reflect nearby activities in individual populations. It has become evident that in addition to resolving a number of genuine questions about the FPs (Bédard et al., 2004; Reimann et al., 2013; Anastassiou and Koch, 2015; Herreras et al., 2022; Ness et al., 2022), some general concepts need to be fleshed out to bring them closer to the physical principles at play. As such, the recent boost to computational capabilities and the advances in simulation environments has made it possible to explicitly formulate a major problem, that of scaling micro to mesoscopic sources, which along with the dipolar nature of neuronal currents, will define the 3D geometry of FPs (Varona et al., 2000; López-Aguado et al., 2002; Fernández-Ruiz et al., 2013; Reimann et al., 2013; Torres et al., 2019; Herreras et al., 2022).

Until this century, the primary response to the problems caused by remote sources has been to try debugging FPs by eliminating distant contributions, such as for instance current-source density analysis (CSD: Mitzdorf, 1985). CSD helps determining the local or remote origin of FPs in the recorded sites (e.g., Carmichael et al., 2017). However, the CSD is unsuitable for spontaneous FPs by a number of reasons (Herreras et al., 2015), amongst which is the fact that they cannot separate the mixed potentials from strongly overlapping sources near the electrodes (LFPs) to recover reliable time courses for each of them (Martín-Vázquez et al., 2013).

**FIGURE 1:**
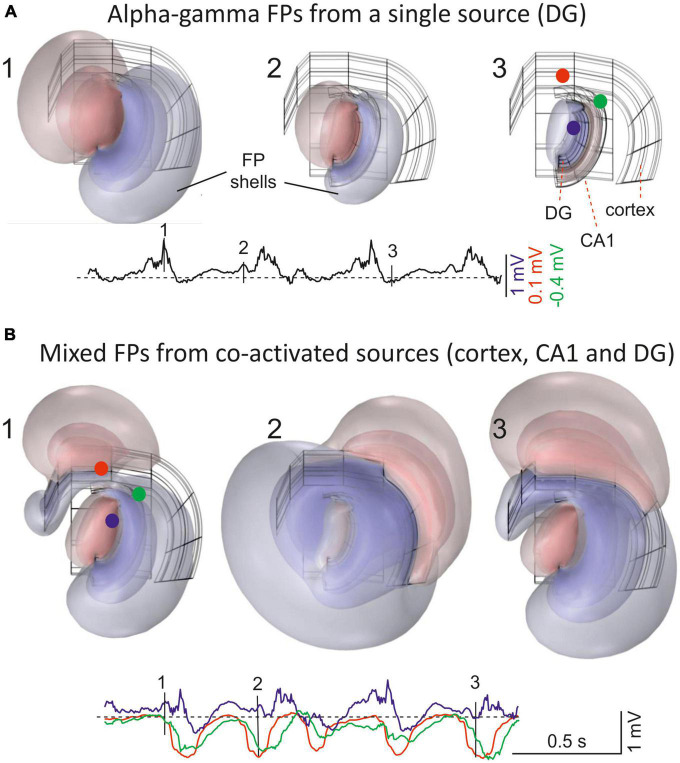
Field potentials (FPs) extend far from their population sources, invading other areas and mixing. **(A)** 1–3 are snapshots of the alpha-gamma activity generated in the Dentate Gyrus (DG). The instants chosen are indicated by vertical dashes in single site customary traces. The 3D structure of the sources is shown in the background in black. These were modeled as contiguous dipolar blocks of current (source and sink) with dimensions and spatial location roughly matching those in the Rat Brain Atlas by Patxinos and Watson (2006). Three pathway-specific sources of current were modeled, a synaptic input to middle dendrites of layer 5 pyramidal cells in the cortex (outer frame), an input to the st. lacunosum-moleculare of the CA1 pyramidal cells (intermediate), and the LPP input to Dentate Gyrus granule cells (inner frame). The colored concentric spheroids represent isopotential surfaces (blue and red shells of FP are negative and positive values, respectively). The lowest level plotted (outermost spheroid) is at ± 0.13 mV. These are from a point in the thalamus (blue) and two in the cortex (red and green) (note different scaling). **(B)** 1–3 snapshots during co-activation of multiple sources in different structures: alpha-gamma in the DG, slow waves in the cortex, and hippocampal theta rhythm in CA1. The model is based on finite-element methods (FEMs) using realistic dimensions, current densities and temporal dynamics (as in Torres et al., 2019). Note that FP shells at different instants maintain proportional amplitude across space when only one source is active **(A)**, but not when multiple sources are co-activated **(B)**. For a dynamic representation see Supplementary Video 1.

Given the highly independent dynamics of different brain sources, the contribution of each to a specific recording site will depend on whether the associated FPs add or subtract from the others, and their relative weight there, establishing a kaleidoscopic of shifting contributions over time and space (Supplementary Video 1 and Figure 1). Conveniently, both the separation of sources and the determination of their local or remote origin can today be achieved advantageously by blind source separation techniques (see below) (Herreras et al., 2015; Whitmore and Lin, 2016; Torres et al., 2019). Before tackling this situation, it is important to understand how each source contributes to the amplitude of a FP and to its rate of decay. We have previously discussed a number of the misinterpretations of FP data that derive from simple recording settings and technical limitations (Herreras, 2016). Also, we offered guidelines to understand mesoscopic FPs from microscopic sources (Herreras et al., 2022). Here we will review recent multisite recordings and model multilevel data, discussing how they help the notions of volume conduction and our understanding of the spatial reach of FPs, more closely aligning these with the principles of physics. We focus on how variables related to single sources (e.g., size, shape, partial activation, uneven current density and dipolar nature) affect the amplitude and rate of decay of FPs. A number of experimental cases are reviewed illustrating how the particular cytoarchitecture of the activated region or structure affects the spatially uneven spread of their FPs and the implications of this for interpretation.

We first briefly introduce some general considerations that will help the spatial aspects of FPs.

### 1.1. How the properties of the medium affect FPs

The propagation and decay rate of electric fields in the brain follow principles that are common to any electromagnetic field. Hence, these features are determined by the characteristics of the source and the properties of the medium in which they propagate (resistive, diffusive, magnetic or inductive). The latter three were considered of little relevance by Lorente de Nó (1947) who proposed the quasi-stationary approach generally accepted as a reliable take on the volume-conductor theory in the brain (Woodbury, 1960; Plonsey, 1964; Rall, 1977; Nunez and Srinivasan, 2006). How the different electrical properties of tissues potentially influence the electrical fields within them has been considered on several occasions and some significant deviations of FP spread from expected in purely resistive media have been found (Elul, 1971; López-Aguado et al., 2001; Bédard et al., 2004, 2022; Holcman and Yuste, 2015; Savtchenko et al., 2017). However, it is widely accepted that the spatial extent of the FPs is not differentially affected within the most common frequency band of interest (0.1−200 Hz) (Nunez and Srinivasan, 2006; Ranta et al., 2017; Ness et al., 2022). Resistivity is not fully homogeneous in the brain and there are significant deviations from a uniform propagation of electric fields in regions where cellular elements of a particular geometry are grouped together (e.g., anisotropy in large fiber bundles), or where there are tissue heterogeneities (e.g., in layers with tight soma packing or near the ventricles: Li et al., 1968; Okada et al., 1994; López-Aguado et al., 2001; McCaan et al., 2019; Torres et al., 2019). These regions are important to bear in mind when tracking sources from recordings at remote sites, e.g., the EEG (Güllmar et al., 2015). Changes of tissue resistivity are also known to be produced during postnatal development, pathology or intense activity (Adey et al., 1966; Hoeltzell and Dykes, 1979; López-Aguado et al., 2001; Makarova et al., 2008), which should be used to correct for recorded FP values and predict their spatial reach. More important, brain sources have complex 3D geometry and are dipolar. It will be appreciated below that the impact of the source geometry on FP amplitude and spread exceeds by large the modulations derived from possible inaccurate appraisal of the mentioned properties of the volume conductor (see discussion in Herreras, 2016).

### 1.2. The pathway-specific nature of mesoscopic FPs: A conceptual leap

In contemporary literature, temporary characteristics of the FP are often used as the main identity card of a neuronal source (e.g., gamma or theta oscillations). The challenge, however, is to identify the anatomical substrates. It is already known that temporal patterns are not exclusive to a single source and that each source may express several patterns (Herreras, 2016; Herreras et al., 2022), further supporting the need for anatomical identification to avoid unfounded generalization of FP mechanisms in different structures.

If we were to find a structural unit of current that could serve as a canon for mesoscopic FPs, we would be in a position to predict the amplitude and spatial extent of the FPs generated by any given source through the use of anatomical information. At the microscopic level it was proposed that one or a small group of synapses (synaptic functional units) form extracellular dipoles from which FPs arise (Elul, 1971). However, the arborized structure of neurons distributes the current in such diverse ways that each synapse produces a unique extracellular 3D voltage shell (Rall, 1967; Behabadi and Mel, 2014; Toharia et al., 2016; Herreras et al., 2022). By the same token, individual neurons can be ruled out as elementary sources, since any single neuron can adopt innumerable geometries of dipolar currents when activated by different synapses, or by groups of them (Herreras et al., 2015, 2022).

**FIGURE 2:**
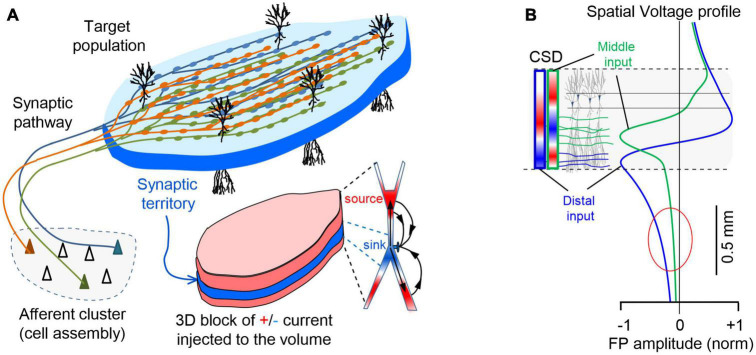
Spatial coherence of FPs is brought about by the pathway-specific nature of the sources. **(A)** The spatiotemporal clustering of currents required to raise a mesoscopic FP is provided by the synchronous firing of a cell assembly projecting onto an orderly population. The scheme illustrates a simple case corresponding to excitatory input from a neuron assembly to a target population with neurons arranged in palisade (e.g., the CA3-CA1 Schaffer pathway). Numerous buttons *en passage* ensure synchronous synaptic currents in the target population and the stratified organization of the synaptic territory can be perceived as an initial anatomical reference for source geometry (pad-like bluish block occupying a dendritic stratum). However, as the inward current at the synapses (sinks, in blue) travels inside neurons, it leaks out back into the volume (sources, in red) through adjacent membrane domains, jointly shaping a conglomerate of sheet-like stacked currents that cover the entire physical space occupied by the population. **(B)** Voltage profiles do not match CSD profiles. Computed voltage profiles for the activation of the Schaffer (green) and Perforant pathways (blue) that establish synaptic contacts in the middle and distal parts of the dendritic tree, respectively. The dashed lines represent the spatial boundaries of the source population within which the extracellular currents arise (colored bars on the left indicate the spatial distribution of currents for each pathway, i.e., the current source density: CSD) along the cell generators that produce the voltage profiles. Note the dipole or quadrupole (sandwich-like) configuration of the currents for inputs to distal or mid-dendritic portions of neurons, respectively. Distal inputs favor the extension of FPs away from the source population (oval).

An invaluable reference at the mesoscopic level was finally attained through the application of spatial discrimination tools to high-density linear recordings in ordered structures (Korovaichuk et al., 2010; Makarov et al., 2010). Algorithms like independent component analysis (ICA, Bell and Sejnowski, 1995; Choi et al., 2005) can disentangle spatially coherent FP components that turn out to be pathway-specific (Fernández-Ruiz et al., 2012; Benito et al., 2014). Anatomically realistic computer models validated this approach (Makarova et al., 2011; Fernández-Ruiz et al., 2013), and led our team to propose a new anatomy-based concept of brain sources (Herreras et al., 2015). These were defined as the coherent extracellular currents produced by a specific neuron population upon synaptic input from a given pathway (Figure 2A). Consequently, the build-up and shaping of spontaneous FPs is similar to that of simple evoked potentials except that firing synchrony of the afferent population is less stringent (Fernández-Ruiz et al., 2012). This concept extends to the source/sink notion by Lorente de Nó (1947), which focused on cytoarchitecture of the population acting as source of current (neuron morphology and population arrangement), and adds an afferent pathway as a third element determining which part of the neurons/population will actually deliver the positive and negative currents to the surrounding volume. Indeed, grouped firing of neurons (cell assemblies) entails the synchronous activation of fibers within a pathway, which provides the necessary synchrony and spatial clustering of groups of synapses in the target population. The individual microscopic fields merge into coherent spatial blocks of FP activity, which in laminar populations peak in the synaptic territory (Benito et al., 2014). Naturally, since most pathways have a distinctive synaptic territory, the corresponding spatial blocks differ and cannot be used as canon. Note that asynchronous inputs in multiple pathways targeting the same population do not enable significant spatiotemporal summation of currents and in fact, they become unmeasurable (Elul, 1971; Łȩski et al., 2013). This concept helps visualize the FP source as a concrete 3D structure, closely matching the territory of a specific synaptic pathway from which FPs irradiate into the volume (see Supplementary Video 1). Also note that because of variable co-activation of multiple pathways one cannot expect a spatially stable structure of FP gradients in the volume and their disentanglement is necessary to access each one’s distribution and time-course (Herreras et al., 2015).

### 1.3. Spatial gradients of voltage give information on the location of sources and the spatial reach of the associated FPs

**FIGURE 3:**
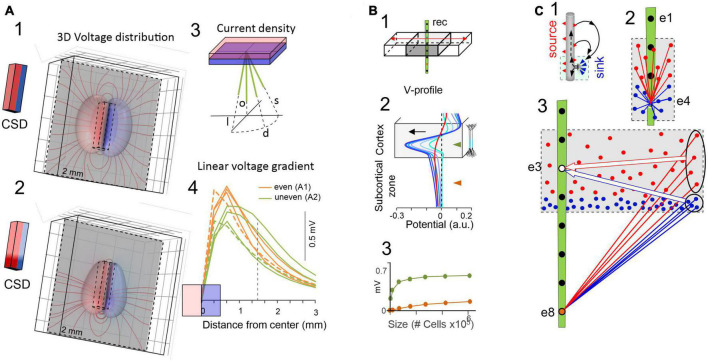
The critical role of source geometry and current density in defining the rate of field potential decay. **(A)** Non-symmetrical dipolar sources produce FPs with different rates of decay. Panels (1,2) show the 3D computation of isopotential spheroids (only three levels are represented) generated by rectangular dipolar sources with the long side doubling the short one (4 mm × 2 mm × 0.5 mm). The lines of current are drawn on a plane perpendicular to the source. In panel (2) the source maintains the same overall geometry and total current density, but this is unevenly distributed. A few linear profiles of the voltage have been drawn (4) to better appreciate the different rates of decay. All plots depart from the center of the block as indicated in panel (3) and scan the volume orthogonal to the dipolar source (o), or they are tilted at identical angles to the long (l) or the short (s) sides, or in between (d). Profiles in orange and green correspond to 3D configurations in panels (1,2), respectively. It can be appreciated that the “mean” distance between the electrode and the source cannot be used to predict the amplitude and spatial reach of FPs. **(B)** Model analysis of the effect of increasing the size of a cortical module (0.5–6 mm) on the amplitude of the FPs recorded inside (gray) and outside subcortical sites (brown). Voltage profiles in panel (2) are colored from cyan to dark blue for enlarging cortical modules. The curved (in-source) and the flat portions (out-source) of the voltage profiles grow at different rates (3). Whereas the curved portion is mostly contributed by nearby neurons, the flat part can be said to be contributed by neurons at a distance from the recording tract. This distant contribution (red profile) can be computed by excluding neurons close to the electrode [neurons within 1 mm of the electrode were excluded in the red profile of panel **(B)** 2]. This is schematically explained in panel **(C)**: Panel (1) is a scheme of the activation of an excitatory synapse and the resulting microscopic sinks and sources (blue and red arrowheads) on the outside, each of which raises a potential that integrates into recording sites weighted by distance. For small sources (2) the different distance of “point-like” sources or sinks determines the amplitude and polarity at electrodes inside the active population. For large sources (3) the distances of distant sources and sinks equalize at electrodes inside the population, largely neutralizing each other (large arrows), but less so for electrodes at a distance from the population (ovals). Although each “point-like” current has a small impact, the large numbers build significant potential over a distance. Thus, distance becomes irrelevant when many fields are combined and their joint geometry takes over [**(B)** reproduced with permission from Torres et al., 2019. **(A)** Computed with the same model].

In examining the 3D voltage distribution of specific brain sources using realistic models, the spatial decay of FPs can be seen to vary in different directions, as reflected by the irregular spheroids formed by isopotential surfaces (Figure 1 and Supplementary Video 1). Sampling of these volumetric shells with linear arrays renders spatial voltage profiles that may vary along the source according to its geometry and the orientation of the array (Figure 3A). These profiles are important as they can also be achieved in experiments and provide a common tool to benchmarking the cellular and subcellular mechanisms. Thus, an ICA applied to multisite linear FPs returns a limited number of spatially coherent components, each characterized by a stable and unique spatial voltage profile (Makarov et al., 2010). Such single-source spatial profiles are time-invariant and they reflect the relative power along the recording array of the potentials elicited by an activated pathway (Figure 2B). Importantly, voltage profiles provide a stationary view of the reach of the FPs in the volume. For instance, accelerating curved spatial gradients indicate that the recording array is inside or close to the source, whereas non-zero, flat profiles correspond to remote sources. The latter phenomenon is explained by the small decay of potentials far from the source, maintaining similar amplitudes at all sites in high-density linear arrays. However, more complex spatial profiles can also be found, particularly for sources in curved structures (e.g., the hilus of the DG), and interpreting these requires anatomic guidance (Fernández-Ruiz et al., 2013).

One might expect that the spatial voltage profile would drop-off toward zero as it becomes distanced from the source. Such patterns have been found for several but not all hippocampal and cortical sources (Benito et al., 2014; Torres et al., 2019). In fact, spatial profiles often exhibit a curved segment and a flat tail with smaller amplitude that expands many millimeters from the source (Figure 3B), even covering the entire brain (e.g., slow cortical waves). The curved and the flat components can be experimentally detached from each other by the blockade of activity near the recording array. Detailed biophysical models indeed show that both spatial components belong to the same source (Torres et al., 2019), whereby the curved part is driven by the neurons closest to the recordings inside the source and the flat part by recordings outside and more distant (Figure 3C). In turn, the absence of decaying tails in a spatial voltage profile indicates that the generating sources do not extend significant potentials into other structures.

### 1.4. Dipolar neuronal currents make the mean distance to the source irrelevant as geometry prevails

There is a widespread notion that the distance from the source to a recording site determines the amplitude of the FP. However, when referring to brain sources, the generic use of the term distance is misleading since the sources are varied, heterogeneous and most importantly, dipolar. The mean distance from the source to a recording site does not explain why most brain structures do not contribute significant FPs despite their neurons being strongly activated. Similarly, it does not explain why different pathways elicit FPs in the same population that spread distinctly in the volume, or why a single pathway produces FPs with different rates of decay away from the same target (source) population.

Some cases have been illustrated in which the FP at a given distance from the center of the activated population acquires totally different values in different directions (Figure 3A). Rather than being caused by anisotropic media, this phenomenon is due to the 3D nature of the source and as such, the current density at all points within it needs be weighted at the chosen recording site to define the single-point voltage measurement. Indeed, some parts acts as a source (positive) and others act as sinks (negative), and the current density is likely to be non-uniform in each and with some parts closer than others (for an introduction to volume averaging of microscopic currents see Herreras et al., 2022). The mesoscopic dipoles remaining after cancellation of positive and negative miniature currents still retain a complex 3D geometry with uneven current densities (Figure 3C). Therefore, we should refer to favorable or unfavorable source geometries rather than to distance. This is not merely a semantic distinction, as the latter entails a simplification that supports what is often considered an insignificant impact of distant sources on FP recordings.

From the above considerations and given the complexity of brain sources, *the amplitude of a FP at the origin does not determine how far it reaches*. As such, rather than the peak magnitude of a current it is the overall spatial distribution and density of its sources and sinks that defines the rate of spatial decay of the potential. In some cases, this will be determined by the extension (size) of the activated population (Figure 3B) and/or the arrangement of neurons in space, whereas the location of the inputs may be the primary factor in other cases (Figure 2B). Thus, inputs to distal or middle portions of a dendritic tree produce far-reaching and mostly localized FPs, respectively, consistent with the overall dipolar or quadrupole configuration of the unbalanced electric sources and sinks in the volume (Herreras, 2016). This is relevant to identify which pathways produce FPs in one structure that will be exported to others. For instance, the lateral perforant pathway (LPP) input to distal dendrites of granule cells (distal input ≈ dipolar configuration) provokes potentials that reach the adjacent CA1 field and even the cortex, where they mix with FPs from local sources. By contrast, those FPs associated with activation of the medial perforant pathway (MPP) or Schaffer fibers (middle input ≈ quadrupole configuration) remain circumscribed to the Dentate Gyrus (DG) or the CA1, respectively, (Fernández-Ruiz et al., 2013; Benito et al., 2014). These geometrical constraints explain why hippocampal alpha waves may penetrate far into the cortex and other brain regions, whereas hippocampal sharp waves or gamma waves have a more limited spatial spread (Torres et al., 2019).

## 2. Local vs. volume-conducted field potentials: An ill-posed dilemma

At any instant, multiple sources are co-active in the brain. The global picture is that of a 3D space in which static regions act as variable sources of current and FPs are produced, exported and mixed in the volume conductor. The compound electrical field generated extends as a continuum through the volume of the brain, and it has space-varying amplitude according to each source’s location and rate of decay (Supplementary Video 1). The regions interposed between active sources still incorporate potentials from several others and these volume-conducted potentials also invade active sites, contaminating the FPs generated there. Consequently, whether a site hosts one, several or no sources, the FPs there will be a mixture. Indeed, the reputation of volume-conducted FPs as contaminants puts the spotlight on where we place the electrodes to minimize their impact, but this desirable goal is rarely pursued in practice, and may not be possible anyway (see below). Such contamination may confound measurement of waveform parameters (e.g., amplitude, phase, duration) and the frequency content of longer epochs (Herreras et al., 2022). Actions to minimize this problem typically involve multisite recording settings that enable nearby electrodes to be differentiated and off-line analytical removal through CSD analysis (Lorente de Nó, 1947; Leung, 1979; Mitzdorf, 1985). Modern approaches as spatial discrimination techniques (e.g., the ICA) do not remove any activity; rather they disentangle all sources and provide each one’s site of origin directly from the spatial profiles (Herreras et al., 2015).

Importantly, this “contaminating” idea by volume-conducted FPs has diverted attention away from the key fact that even after remote contributions are removed, the so-called local FPs (LFPs) are themselves mixtures, constituted of several close, or even overlapping sources. One might therefore consider that all FPs are mixtures of mutually contaminating sources, although this is an impractical notion and rather, we prefer to retain the more stringent terms of source co-activation and FP blending or mixture. Note that FPs recorded inside or outside of sources are not a strict parallel of local and volume-conducted potentials, since FPs are never local and all FPs are volume-conducted. Thus, we next discuss the distinguishing features of FPs in these two regions that help identify them in experimental recordings.

### 2.1. Field potentials originated in remote sources are ubiquitous and show distinguishing features

In all the brain structures explored to date through multisite recording and spatial discrimination techniques (e.g., the ICA), an FP component with non-zero flat spatial profile has been found (i.e., potentials that originate at sources remote to recording sites). By contrast, only a few structures yield FP components with curved spatial profiles (nearby sources) (Herreras et al., 2015). The relative variance of remote contributions is rarely below 5−10%, and it climbs to 100% in structures whose cytoarchitecture is not geometrically fit to generate mesoscopic sources of current (Martín-Vázquez et al., 2016). To gain an idea of how much voltage is introduced by sources beyond the recording position, it is helpful to compare differential (bipolar) to monopolar (grounded) recordings (Elul, 1962). The former typically show FPs about 10 times smaller than the latter (Gómez-Galán et al., 2012) and thus, most of the activity captured in FPs is remote rather than local. Several experimental studies indicate that FP activity originates from neurons circumscribed to a radius of a few hundred microns (Katzner et al., 2009; Liu et al., 2015). Actually, the rate of decay is specific to each source and cannot be generalized (e.g., Figures 2B, 3A). As a case in point, recent quantitative studies found that hippocampal theta and gamma rhythms in the rat brain may extend millimeters away from their sources, and these potentials have a large enough amplitude at distant sites to supersede local activity (e.g., the visual cortex: Torres et al., 2019). Other structures display FPs that are almost completely of a remote origin, such as in the striatum, the lateral septum and the habenula (Martín-Vázquez et al., 2016; Lalla et al., 2017; Bertone-Cueto et al., 2020). Detailed models confirmed that multipolar neuron morphology and/or the scattered arrangement of neurons in these structures does not favor the build-up of extracellular current sources and sinks, and hence FPs, as originally proposed by Lorente de Nó (1947). Recordings at different sites within structures with such unfavorable anatomical elements exhibit nearly identical FP fluctuations, as there are no local contributions to establish any spatial voltage gradients. Such cases are well detected by the ICA technique, which will only return components with flat spatial voltage profiles, i.e., all activity is volume driven from remote sites (Martín-Vázquez et al., 2016).

It then appears that so-called volume-conducted potentials are rather ubiquitous, and that they are likely to contribute to the waveform parameters of individual waves and the frequency content of long epochs anywhere (Herreras et al., 2022). As such, these potentials will introduce unknown error in quantitative studies that address functional connectivity using single electrodes. It should be noted that remote sources cause self-coherence when FPs are recorded in different structures (Florian et al., 1998; Torres et al., 2019).

**FIGURE 4:**
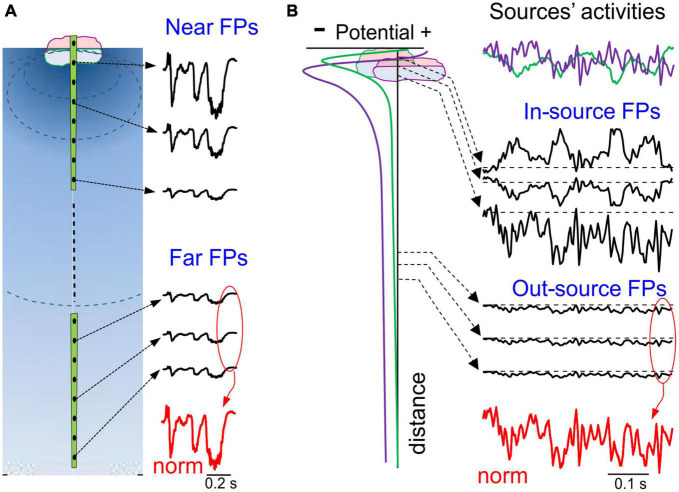
Spatial and temporal ambiguities of potentials generated by local and remote sources. **(A)** Potentials decay fast close to the source and slowly away from it, a feature that is used as an arbitrary segmentation of the volume to circumscribe “local” and remote FPs. FPs elicited by a single source have an identical time course throughout the volume (normalized at the bottom in red), only varying in amplitude or polarity at different sites. **(B)** A case of two overlapping sources in the same structure (two synaptic pathways): left column, spatial voltage profiles; right column (from top to bottom), color coded activities of the two sources (top); in black, mixed FPs at different distances; at the bottom, normalized potentials of distant sites. Note that close to the sources the time course is strongly site-dependent, whereas at positions further away time features are invariant and exhibit blended time marks.

Since distant co-active sources contribute to remotely generated potentials, the time course of each cannot be discerned. In this sense, such potentials are as poorly defined as raw FPs or scalp EEGs, and they do not help discriminate whether they originated from one or many sources (Figure 4, red tracings). For instance, the FPs recorded in the lateral septum of anesthetized rats turned out to be entirely made up of remote contributions, and with temporal features reminiscent of both cortical and hippocampal sources (Martín-Vázquez et al., 2016). Also, FPs recorded in the striatum are entirely of a remote origin and they are a mixture of slow waves volume-conducted from the cortex, on top of which occasional bouts of alpha waves arise that can be traced to the hippocampus (Lalla et al., 2017; Torres et al., 2019). Identifying the structure of origin of remotely generated FPs is not trivial, although placing the test electrodes in the suspected sites, or even better, actively exploring the volume with moving multisite arrays may help.

A distinct form of FP contamination, which also enters all recording sites in high density arrays with similar power, occurs when the reference electrode is placed at sites that pick up FPs, but this has no direct relation to volume conduction (Fein et al., 1988).

### 2.2. Features of not so local LFPs

Several pathways commonly converge on a given neuronal population and unless the synaptic territories match tightly, each pathway sets distinct zones of positive and negative current, i.e., the geometry of the respective dipolar sources differs even when arising from the same population of neurons. All regularly arranged structures so far explored through spatial discrimination techniques reveal multiple overlapping FP sources with distinct profiles along the somatodendritic axis of source neurons, which includes the cortex and hippocampus of rodents, and the lateral geniculate nucleus of monkeys (Benito et al., 2014, 2016; Makarova et al., 2014; Torres et al., 2019). Moreover, unpublished data from our lab includes the superior colliculus and the olfactory bulb of rodents in this category.

From a practical point of view, since each afferent pathway has its own dynamics, merging associated potentials into FPs diffuses the individual temporal patterns and hence, the waveform parameters become unreliable (see Figure 4 for an illustration of some aspects of this problem) (Martín-Vázquez et al., 2016). Steep gradients within the source population that decay outwardly in a pathway-specific manner are an important characteristic of FP mixing. In some regions of the brain, particularly in layered structures, each source’s relative contribution may vary considerably when the recording position is displaced only a small distance due to the strong stratification of the synaptic pathways. Hence, the temporal fluctuations in the mixed FP reflect the markedly different proportions of each pathway at different sites. In the hippocampus, spontaneous FPs may display large differences at sites as close as 100 microns (Benito et al., 2014) and in this regard, visual inspection of multisite linear recordings is sufficient to reveal smooth or strong spatial gradients. Spatial breakpoints can even be seen in recordings only 50 μm apart through the abrupt differences in the respective temporal features. These sites are generally associated with anatomic boundaries, such as the limits of two neuron populations, cell body layers or the demarcation of synaptic territories, which can help identify and/or localize the sources. As the distance to the sources increases, the spatial voltage gradients smooth out and flatten, such that their relative contribution stabilizes (Figure 4B).

As noted above, brain sources are not static entities and the volatile activation of synaptic pathways means that the spatial reach of FPs varies continuously. As such, a given recording period will contain remote and nearby sources that contribute to a varying degree over time. For instance, the amplitude (voltage) of any FP motif may vary with: (a) the proportion of neurons in a population activated that occupy stable spatial boundaries (fixed geometry); or (b) by additional recruitment of active neurons at more distant sites (varying geometry). In the latter case, since an electrode cannot provide information on the variations in the source, one may erroneously interpret a change in waveform parameters as reflecting the activation of different populations of neurons. In large structures, the extra voltage contributed by distant portions should be considered *sensu stricto* a remote contribution (“self-contamination”). The only way to overcome this uncertainty is by simultaneously recording from different parts of the same anatomical source.

## 3. The variable reach of field potentials from extended sources

Let us now review the experimental and modeling data that indicates how source geometry influences the amplitude and spatial extent of FPs originating in brain structures that are more favorable to FP build-up, such as the cortex and hippocampus. Orderly structures like these promote a segregation of the sources and sinks within their volume during the coherent synaptic activation of neurons, which is due to reduced cancellation among their neuron units. They are also optimally suited to spatial discrimination techniques that can reveal their multiple and often overlapping FP sources or generators. The voltage profiles obtained through these techniques reflect the spatial distribution of the FPs and offer a means to further investigate the relevant anatomo-functional mechanisms by matching them to those obtained in large-scale studies of anatomically realistic models. In this sense, a number of biophysical models have been deployed with sufficient anatomical and functional variables to provide time-varying FP gradients in 3D. These spatial gradients can be compared to experimental ones and they help identify the anatomical elements that produce them. Finite element methods (FEMs) and anatomically realistic (compartmental) neuron models can offer similar efficiency in the feed-forward reproduction of FPs in a volume conductor (Fernández-Ruiz et al., 2013; Torres et al., 2019; Ness et al., 2022), provided that the macroscopic sources used in the former accurately reproduce the spatial distribution and density of the currents (not the anatomy). Less realistic model approximations are not suitable as they cannot adequately discriminate suitable from unsuitable geometries of co-activated neurons and populations.

On the experimental side, some recording devices have been developed to gather 3D data (e.g., 3Dmatrix: van Daal et al., 2020), although they are still to be manufactured in a way that they can be scaled to obtain the entire 3D voltage shell of potentials elicited by brain sources that are too large or too small. By default, spatial samples of the voltage gradients (Figures 2B, 3A) can be obtained by exploring the volume with linear arrays (Benito et al., 2016; Ortuño et al., 2019; Orczyk et al., 2021). The instantaneous depth profiles of raw FPs vary continuously in concordance with the ongoing activation/deactivation of the sources. However, blind source separation techniques have proven efficient in separating these (Makarov et al., 2010; Herreras et al., 2015; López-Madrona and Canals, 2021).

The results reviewed pertain to geometrical conditions of the source that condition the interpretation of FP waves recorded inside the source volume (self-contamination), while also potentially highlighting the strong contribution and even dominance of FPs in other regions.

### 3.1. Field potentials from small cortical modules: Lateral cortico-cortical “contamination”

**FIGURE 5:**
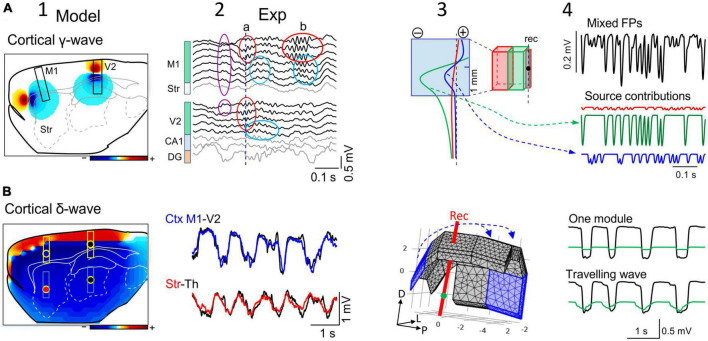
The reach of cortical FPs depends on the specific geometry of the sources. **(A)** Computational (1) and experimental findings (2) on the activation of small cortical modules (0.5 mm) of layer 5 pyramidal cells during gamma waves in two cortical areas (M1 and V2). The computation is performed over the entire hemisphere (3D) but only one sagittal cut is shown with contour plots of the potential at a chosen instant. Scale color bar: ± 0.15 mV. The boxes mark the sites where recording arrays were placed in experiments to capture laminar FPs in panel (2). The limited spatial extent of gamma bouts in experiments is noted by the lack of coherence between the waves in different areas (a) or the region-selective occurrence (b). Other gamma waves (purple oval) appear with invariant waveforms along the cortical width and even in the striatum, indicating an extracortical origin of a gamma source that extends into large brain areas. Panels (3,4) show a different computer analysis of cortical blocks with independent dynamics and different sizes. It can be appreciated how small cortical modules (in blue) can exhibit FPs dominated by nearby larger modules (cortico-cortical contamination). The black trace corresponds to the FP mixture in the small module and the colored traces are the input activities to each module. The voltage profiles (3) and the time course of mixed FPs (4) show the greater contribution of near modules in red and green than its own. **(B)** (1) Similar computer simulation as in **(A)** for slow cortical (delta) waves spanning large portions of the cortical mantle predict that it reaches all sites in the rat brain with large amplitude (Scale color bar: ± 0.6 mV), which is confirmed in experiments (2). Actually, the amplitude reduces only moderately in subcortical sites far from their origin (the recording sites are marked by colored dots in “1”). Panel (3) illustrates the 3D representation of about 2/3rds of the cortical mantle used for the computation. Assembled rectangular blocks of dipolar current were used (numbers are mm). The blue arrows mark the direction of the slow wave traveling across the cortical modules. Upper traces in panel (4) represent the activation of a single cortical block, and the lower traces depict computed potentials during sliding activation (100 ms delay between contiguous cortical blocks) of the entire cortex [the first and last block are marked in blue in panel (3)]. (Columns 1 and 2, and panels **(A3,A4)** are adapted from Torres et al., 2019).

The neocortex is the largest structure in the mammalian brain and although it has a fairly well-maintained internal columnar organization, the area-specific afferent and associational circuitry establishes strong regionalization that defines discrete functional and operation modules (Arcaro and Livingston, 2021). Thus, the cortex is typically activated in a patchy pattern that reflects processing demands in behaving animals (Frostig et al., 2008; Çelik et al., 2021). In this state, functional cortical areas appear as small modular sources that process specific sensory cues, or information from body parts or related to cognitive tasks (Figures 5A1, A2; Katzner et al., 2009; Liu et al., 2015; Rogers et al., 2019). Biophysical models have shown that the size of different cortical areas determines how far cortical FPs reach into adjacent cortical areas (Figures 5A3, A4; Torres et al., 2019). In anesthetized rodents, some but not all of the alpha and gamma cortical generators extend through limited portions of the cortex, and the associated FPs reach a little beyond the physical boundaries of the respective modules (Figure 5A2). That is, the pronounced rate of voltage decay away from the source minimizes cortico-cortical contamination between adjacent modules.

Since the degree of contamination depends on the relative size of active modules, the smaller the cortical module the greater the contamination of its FPs by adjacent ones (Torres et al., 2019), even to the point that they may be overshadowed by this contamination (Figures 5A3, A4). This fact must be taken into consideration when studying receptive fields or exploring the relationship between cortical regions. Note that since cortical modules may be activated in different epochs, the ongoing changes in wave parameters can be attributed indistinctly to local changes of excitability or to sporadic lateral contamination from adjacent modules. For example, different thalamo-cortical tracts activate similar synaptic territories of homologous cells in different cortical areas (Figure 5A2; Haegens et al., 2015). When nearby cortical modules emit fast rhythmic activities at a similar frequency, it may be anticipated that the resulting time-related parameters of gamma waves will be severely distorted in the merged FPs unless they are in phase (Herreras et al., 2022). Therefore, determining the extension of the functional modules of interest and that of their neighbors is essential. Anatomo-functional studies have outlined a number of cortical areas in different species, although it remains unclear if each of these serves as an independent source of current during spontaneous activity. Biophysical feed-forward models of synthetic FPs show that spatial discrimination techniques would be very effective to untangle the contributions of nearby and remote sources (Figures 5A3, A4).

### 3.2. Lateral contamination of FPs between adjacent areas is source-specific

Electrophysiology experiments show that each cortical region harbors several co-localized FP generators with distinct layer profiles (Martín-Vázquez et al., 2018; Ortuño et al., 2019; Orczyk et al., 2021). Since the geometry of the sources is defined by the topology and extent of the coherent synaptic inputs, the FPs of each may have a distinct lateral extension (Benito et al., 2014). The degree of lateral contamination between adjacent cortical areas is not expected to be area-specific but rather, source-specific. That is, synaptic inputs to some neuron types in the columnar circuits produce FPs that may spread more laterally than others. A laminar-specific extension of FPs and interlaminar coupling of currents has been seen in the cortex (Xing et al., 2009; Sotero et al., 2015), which is best explained by source specific geometric features, such as: (1) the range of horizontal connectivity; (2) the degree of stratification of the different pathways; (3) the subcellular location on the target neurons; or (4) their axial or multipolar anatomy.

### 3.3. Coherence across the cortical mantle means cortical FPs can reach everywhere subcortically

During slow wave sleep or under deep anesthesia, large portions of the cortex display highly coherent activity (e.g., slow waves, delta waves or Up-Down states: Destexhe et al., 1999; Riedner et al., 2011; Sánchez-Vives et al., 2017). Macroscopically, slow cortical waves travel across the tissue and several may even co-exist in different sectors of the cortical mantle (Massimini et al., 2004; Bernardi et al., 2018). The mechanisms underlying the synchronous and sliding activation of such large numbers of contiguous neurons across the cortex have been studied intensely but are yet to be fully defined. Coherent activation makes the myriad of individual neuron sources behave as an oversized single current source whose overall geometry and density (CSD) determines its spatial reach. The resulting potentials have an impact on adjacent and distant cortical areas, increasing the proportion of remote sources that contribute to FPs even at very distant sites (red profile in Figure 2B). A distinctive feature of such FPs is that the chemical blockade of synaptic activity near the recordings will produce a mild reduction in the amplitude of the FPs, whereas they will disappear rapidly if they arose from small cortical modules (Torres et al., 2019).

The most noticeable impact occurs at subcortical sites. For example, 1−2 Hz (delta) cortical waves with only moderate amplitude decay (≈50%) can be recorded in the striatum, thalamus, hippocampus, and any other small nucleus down to the base of the brain (Figure 5B2), mixing with local waves. These findings comply with theoretical studies that long ago showed that FPs in curved structures spread preferentially toward the concave side (Woodbury, 1960). When recording from subcortical structures with single electrodes, the large amplitude of such remote slow waves (up to 0.4−0.5 mV in adult rats) might lead one to consider they are nearby, an error that can be avoided through careful exploration of the spatial voltage gradients.

### 3.4. The different reach of FPs from co-localized sources may be paradoxical due to competing geometry-based influences

The combination in a given area of several sources from different cell populations, or their origin in the same neuron population, does not mean their respective FPs will have the same rate of spatial decay and reach, as other geometric factors may be at play. For example, simultaneous recordings at separate cortical sites show different voltage fluctuations riding on top of the slow waves (Mollazadeh et al., 2011; Volgushev et al., 2011; Torres et al., 2019). During the active phase of slow waves some areas may display alpha-beta oscillations that are not appreciated in others, or bouts of gamma waves may appear in one or two different areas that may or may not be in phase (Figure 5A2). These more rapid activities belong to smaller cortical modules than those involved in slow wave production and they may therefore not spread far subcortically. That is, the large cortical area activated during a slow wave contains smaller areas that are apparently activated independently within its physical boundaries.

Numerous studies have shown that many types of neurons can sustain different firing regimes, based on alterations to the rate and/or correlation of synaptic inputs. Such different input patterns can readily appear in FP dynamics, which in monolayered structures like the hippocampus may be ascribed to a single cell type capable of generating significant FPs (Makarova et al., 2011). We previously addressed this capacity of individual neurons in biophysical models (Herreras et al., 2015) and we found it to be caused by the pathway specificity of the FP generators. In experiments, we found that the laminar coverage and spatial extent of a source is determined by the topology of the afferent pathway, which determines spatial modules of coherent activity (i.e., the geometry of the source (Benito et al., 2014). It follows that an individual neuron, which is a geometrically stable entity, can act as geometrically different sources, as many as it receives synaptic pathways. This can be considered a general principle that can also be applied to the cortex, although its multilayered structure complicates the identification of cell and input pathways to individual sources (Ortuño et al., 2019; Torres et al., 2019). The different extensions of the sources will have notable consequences on the reach of their respective FPs into subcortical sites (compare Figures 5A1, 5B1). For example, while extended sources can be expected to produce far-reaching FPs, this can be counteracted by the distribution of the pathway input responsible for it over the cell, or by the morphology of the source neuron itself (Herreras et al., 2015, 2022; Martín-Vázquez et al., 2016). For the time being, these effects remain in the theoretical realm, as the reach of small or area-specific cortical FPs has been poorly addressed in experiments.

### 3.5. Giant FPs in the hilus are not local FPs

**FIGURE 6:**
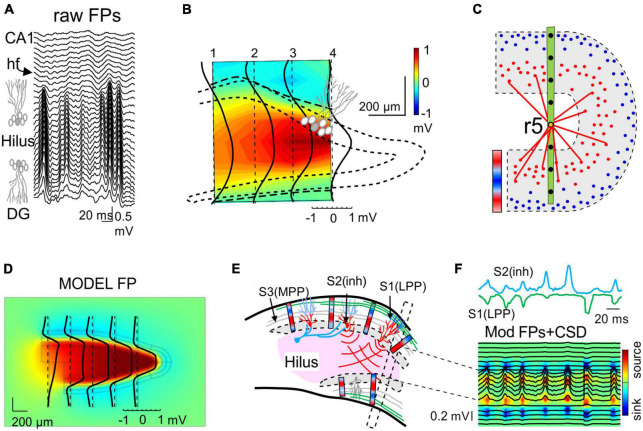
Population curvatures promote greater FPs out of the physical space of the active neurons: the Hilus of the Dentate Gyrus. **(A)** Laminar recordings across the Dentate Gyrus (DG): synaptic inputs to granule cells (GCs) of the DG produce giant FPs in the Hilus between the folded layer of GCs. **(B)** Spatial display of the relative power of the medial perforant path (MPP)-specific FPs detached mathematically from other inputs. The dotted line outlines the GC body layer and the dashed line marks polarity reversal (zero value). The large positive potentials (yellow-red) in the Hilus (out of GC layers) outgrow the negative potentials in the synaptic layers 10–20-fold. The black lines are voltage profiles across different recording tracks. **(C)** Explanatory scheme of the potentials associated to an excitatory lateral perforant path (LPP) input in distal dendrites. Blue/red bar indicates the current density across GC layers: while the microsinks scatter in the outer dendritic layer (blue dots), the folding causes all the microsources (red dots) in both layers to cluster and approach the Hilus, fostering the combination of their potentials with respect to that of microsinks. The red lines indicate the similar proximity of the latter at the ends of both leafs to an electrode located in the middle of the Hilus (r5). These would be farther away if the GC layer were flat. Although the same can be said for microsources, the smaller average distance of the former has a greater impact. The bar represents the current density across the GC layers. **(D)** Model reconstruction of the relative power of FPs across a sagittal cut of the DG generated by synchronous MPP input to both layers (compare to **B**). **(E)** Scheme showing the cellular dipoles (blue/red bars) representing GCs receiving LPP input (S2) and somatic inhibition (S1). Note that the synaptic territories at both ends of GCs and opposing currents for somatic inhibition and dendritic excitation makes the respective dipoles orientate similarly. The dipoles in the upper and lower blades face each other and project with the same polarity (positive) toward the hilar region. The MPP input (S3, in gray) produces a quadrupole current distribution (not illustrated) that limits the spread of the potentials. The black box depicts the array recording area for traces illustrated in panel **(F)**. **(F)** Model gamma oscillations elicited by a LPP excitatory input (S1, green) and soma inhibition (S2, cyan). The model was fed with source activities (colored traces) obtained from experiments to simulate FPs (black traces). The source/sink currents (CSD) generating the FPs are limited to GC layers, but they are absent in the Hilus. Note that all gamma waves recorded across the Hilus have similar polarity, whether contributed by one or the two inputs, and hence they could not reveal the synaptic origin [Panels **(A,B,D)** are taken from Fernández-Ruiz et al., 2013].

A remarkable case in which the architecture of a population defines the way FPs mix, and how far they reach, is the folding of the granule cell layer in the DG. Experimental examination of FPs in this curved region and realistic feed-forward computations have provided important lessons. First, the synaptic dendritic currents in the outer layers create dipolar curved sheets of current and a dramatic concentration of FPs in the interposed hilar region beyond the source granule cells, increasing up to 20 times in size of the value in the synaptic zone (Figures 6A, B, D; Fernández-Ruiz et al., 2013). This striking and yet paradigmatic case demonstrates that the amplitude of a FP is not sufficient to infer the location of the source of the current, and that anatomical knowledge of the candidate source is required.

The second message drawn from the analysis of FPs in this curved region concerns the use of the polarity of waves as an indicator of the chemical nature of the synaptic pathway. It is widely believed that negative and positive waves reflect depolarizing and hyperpolarizing events, respectively. However, this viewpoint does not conform to the dipolar nature of neuronal currents that run across the membranes twice, forming inward-outward transmembrane loops. It also ignores the influence of remotely generated FPs on time-dependent wave parameters, even on their polarity. Both excitatory dendritic currents and somatic inhibitory currents onto granule cells create similarly oriented current dipoles (Figure 6E). Thus, these two currents contribute positive FPs in the interposed hilar region and they become undistinguishable (Benito et al., 2014; Figures 6E, F). Note that the cells in this region are polymorphic and can be ruled out as significant contributors to FPs. Positive hilar potentials are confined to the physical limits defined by the folded layer of the granule cell somata, as if they could not spread beyond the cell layers. Experiments and computer modeling show that this peculiar distribution is the result of a fine balance of zones with positive and negative potentials, these elicited by each of the two cell layers with opposing dipoles (Fernández-Ruiz et al., 2013). Thus, the blockade of synaptic activity in one blade reveals the full extension of the positive FPs generated by the other blade, which extend beyond the inactive blade and further into the CA1. These findings remind us to stay alert to the fact that mesoscopic sources result from the coalescence of coherent microscopic (cellular) sources (Figure 6C), and that their spatial boundaries result in complex spatial blending of positive and negative fields that arise from the favorable cytoarchitecture of the active group of cells but reach far beyond.

### 3.6. The banana-like hippocampus: Cell packing, stratification and curvatures favor the spread of FPs over a long distance

Each sub-field of the hippocampus is populated by a dominant cell type that displays an optimal geometry for the production of FPs. These principal neurons (pyramidal or granule cells) are tightly packed and arranged in a palisade, which simplifies attributing the cellular origin of the FPs to a single neuron population in each sub-field. However, each sub-field houses several sources that correspond to afferent pathways. These are strongly stratified and although their spatial proximity suggests heavy mixing of the associated potentials (i.e., mutual contamination of waveforms), the sharp spatial gradients facilitate the mathematical separation and identification of pathway-specific generators (Makarova et al., 2011; Benito et al., 2014). The identification of these gradients has contributed enormously to achieve experimental confirmation of the theoretical predictions of the geometric features of these sources and their capacity to generate FPs, and how far these will reach.

Like the cortex, the hippocampal activities that favor far-reaching FPs are those that are coherent over large extensions, like theta rhythms. The cell and network mechanisms underlying this activity have remained elusive for decades and new hypotheses continue to appear (Buzsáki, 2002; Lubenov and Siapas, 2009; Kang et al., 2015; Chatzikalymniou et al., 2021). The presence of multiple intrahippocampal theta generators has been acknowledged (Montgomery et al., 2009; López-Madrona et al., 2020) and they have also been reported in other regions. Indeed, respiratory rhythms of similar frequency were recently said to originate in the DG and other sites (Nguyen et al., 2016; Tort et al., 2018). Such a wide variety of potential contributing sources adds to the confusion regarding the intra- and extrahippocampal generators, and their drivers. Here we limit the discussion to the spatial extension of the former and some associated phenomena.

**FIGURE 7:**
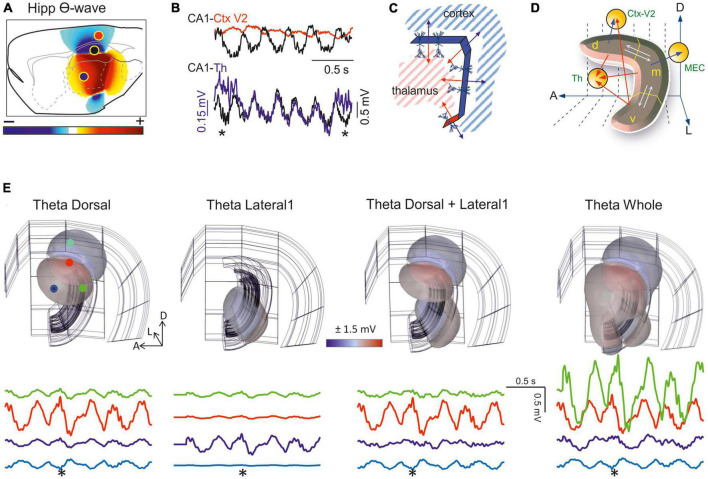
Field potentials (FPs) arising from whole-structure geometry: the banana-like hippocampus. **(A)** Snapshot of the FP spatial distribution over a sagittal cut of the rat brain during activation of theta sources in the stratum Lac-Mol of the CA1 hippocampus. Model FPs have been AC-filtered to remove the strong baseline. The curved shape of this structure fosters the addition of fields toward the inner side (thalamus). This is better appreciated in the 3D representation (see Supplementary Video 2). **(B)** Experimental traces recorded by pairs during theta activity [sites indicated in panel **(A)**]. Note the different amplitude or polarity and the strong but incomplete pairing of the waves at the sites. **(C)** Scheme of the model. The CA1 was built as assembled dipolar blocks of current with the dimensions and current densities obtained from experiments. The overall curvature means the cell dipoles are arranged radially toward the thalamus, such that their potentials cluster in the center and decay much less than expected for planar structures. The double-headed arrows indicate the orientation of the dipole blocks. Note that the cortex and thalamus must receive potentials of opposite polarity. **(D)** Scheme illustrating possible relationships derived from experimental observations on regionalized activation of theta sources along the septotemporal axis. The hippocampus is segmented into three portions, dorsal (d), medial (m), and ventral (v). Theta activation in one or more of these regions may result in a modulation of different theta wave parameters in other segments inside the hippocampus (interactions marked by white arrows) or outside (red arrows). **(E)** Snapshots of the FP distribution in one hemisphere (pseudo 3D representations) during regional activation of the CA1 with theta currents. The structures in the background correspond to the cortical mantle (outer structure), the CA1, and the DG. View of the right hemisphere from the middle line. The CA1 was modeled as four planar blocks of dipolar current arranged as to account for the global curvature. Panels correspond to the dorsal (1), lateral (2), dorsal plus lateral (3), or complete CA1 (4) theta activation. See dynamic display in Supplementary Video 2. Sample FP traces at selected sites indicated by colored dots in panel 1. The asterisk marks the instant used to build the contour plots. Note the different modulations of amplitude, polarity and even the phase of the waves. The regions are activated with a 100 ms delay along the septotemporal axis according to experimental findings. A, D, L: anterior, dorsal, lateral (Same model as in Torres et al., 2019).

Classic recordings already showed that theta FPs appear in the hippocampus and that when they reach adjacent structures they were generally considered to be of remote (hippocampal) origin (Pestche and Stumpf, 1960). Spontaneous theta activity has been observed in many other structures such as the amygdala, striatum, thalamus, nucleus accumbens, habenula, olfactory bulb and parasubiculum, as well as in several cortical regions including the prefrontal, dorsal, entorhinal and cingulate cortices (Borst et al., 1987; Hughes et al., 2004; Siapas et al., 2005; DeCoteau et al., 2007; Glasgow and Chapman, 2007; Rojas-Líbano et al., 2014). Later studies confirmed that some of these regions host genuine theta generators, although the functional connection of these structures to the hippocampus has frequently been assumed without adequate exploration of far-reaching theta FPs. Recent biophysical modeling showed hippocampal curvature to play an important role in the extension of theta FPs into neighboring structures (Figures 7A, B and Supplementary Video 2). Also, synaptic inputs to distal dendrites favor potentials with a strong dipolar moment that decay slowly as they move away. In any case, since there are genuine extrahippocampal theta generators and their spatial extension has yet to be explored, a disambiguation of nearby and remote components is necessary in each structure. As discussed elsewhere the mixing of these generators causes major distortions of local waveform parameters, such as amplitude and phase (Herreras et al., 2022).

The export of FPs to structures other than the generating source itself should not be assumed as a constant influence that modifies (contaminates) the FPs recorded there in a predictable manner. For that to happen the sources must be stable over time, which is never the case. Indeed, behavior dependent changes to coherence between theta activity in the hippocampus and other brain structures have been reported (Tavares and Tort, 2022), which can be explained by independent activation in the two structures or through the regional activation of hippocampal theta waves along the septotemporal extent of the hippocampus, altering FP export. The factors that might influence the theta sources in the hippocampus are more complex than those in the cortex. Thus, the more central position of this structure in the brain, its strong internal and global curvature (folded layers and overall C-shape), and the multiplicity of the theta generators in different subfields (López-Madrona et al., 2020), along with the incomplete spatial coherence (Schmidt et al., 2013), together promote regional differences among theta waves within and beyond the hippocampus. These combinations are too many to speculate, and some of these factors are only just beginning to be recognized and explored. However, they may well account for the large variety of behavioral theta modulations reported in the literature.

It should be noted that if theta potentials reach a site far beyond the hippocampus, they will also affect segments of its own. Hence, the time-frequency characteristics of the theta waves in different hippocampal segments may be contaminated significantly by those from other segments. The true local waveform can only be revealed by using multiple linear arrays and spatial discrimination techniques. Two observations that may be explained by geometric factors should be highlighted. First, the cortical regions above the dorsal hippocampus face the dipolar theta generator that originates in the CA1 sub-field, yet with a polarity opposing that of the structures confronted by concave sites like the thalamus. Thus, theta FPs may have a different polarity in different regions but still belong to one and the same hippocampal source (Figures 7C, D). However, and as mentioned above, conditions are rarely that simple and the differences among the distinct theta generators of the different hippocampal segments at the start/end of their activity may lead to complex modulation of the nearby and remote contributions, resulting in amplitude and phase variations (see Figure 7E and Supplementary Video 2). The time-varying correlations during theta activity typically found between any two recording sites in or near the hippocampus support these possibilities (Hinman et al., 2011).

Another source of waveform discrepancy in sites outside the source arises from a technical drawback, such as the artificial zeroing of the FPs recorded in AC-coupled mode. This renders an artificial succession of positive and negative half waves at a fixed location in space, as opposed to the expected regionalization of the volume in stable positive and negative regions (Brankaèk et al., 1999; Martín-Vázquez et al., 2013). Biophysical models show that theta waves appear to roll over the main hippocampal axis when this technical drawback combines with incomplete coherence (Supplementary Video 2; Torres et al., 2019). In short, studies that use theta waves as a temporal marker, or those exploring the spike-phase relationships or the cellular basis of theta activity, should take in account that the multiplicity of theta sources entails numerous geometrically-born modifiers of time-frequency parameters, including the amplitude and phase of theta waves. Hence, ensuring the geometric stability of theta sources is a must.

### 3.7. Spurious correlations caused by remotely-originated FPs: Cuckoo potentials

**FIGURE 8:**
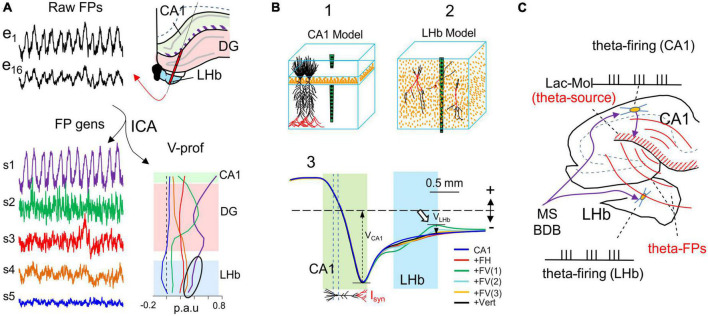
Potentials from far sources may correlate to local neuron firing with no direct relationship: cuckoo potentials. **(A)** The lateral habenula (LHb) is positioned near the hippocampus. Both structures receive theta-pace inputs from the septal pacemakers and exhibit rhythmic theta oscillations in FP recordings (black traces). e1 and e6 are located in the stratum Lac.-Mol. of the CA1 and the LHb, respectively. Spatial discrimination analysis (ICA) of FPs recorded across the boundary between these regions (scheme) highlights a single theta source (S1: purple trace and V-profile) with peaks in the CA1 stratum Lac.-Mol. and DG molecular layer (arrow). However, although the power decays inside and across the LHb (arrowhead), it still makes the strongest contribution there (black oval). Sources S4 and S5 have greater power in the HBl, but they exhibit irregular activity. **(B)** Anatomically realistic model of the CA1 and the LHb. The upper blocks represent the orderly cytoarchitecture of the pyramidal cells in the CA1 and the multipolar tangled cell types in the LHb. The lower plots are the V-profiles obtained for theta inputs (marked in red) onto different cell types (FH: fusiform horizontal; FV: fusiform vertical; Vert: vertical). FV(1–3) correspond to three different subcellular distribution of inputs: only an apical–only input (FV3) produce significant potentials (white arrow). Whereas theta input to CA1 pyramidal cells leads to strong FPs that extend through the LHb, habenular cells barely contributed any FPs (compare contributions V_CA1_ and V_LHb_). **(C)** Scheme explaining the spurious but correct FP-to-spike correlation. Input from the septal area drives neurons in the CA1 and the LHb (purple arrows), both of which fire spikes at a theta frequency. In CA1 theta firing cells activate theta currents in pyramidal cells and large theta FPs, which spread and reach the LHb [Panels **(A,B)** are adapted with permission from Bertone-Cueto et al., 2020].

Ignoring the presence of remote contributions to FPs may result in their assignment to the wrong structure. This problem can be well illustrated if we consider the lateral habenula (LHb: Figure 8), a structure in which neurons receive theta-paced synaptic inputs from the medial septum area that match locally recorded theta FPs, and they also drive theta firing of some nearby neurons (Aizawa et al., 2013). It would seem safe to conclude there is a causal relationship between such matching activities. However, theta FPs in the LHb originate in the nearby hippocampus (Figure 8A; Bertone-Cueto et al., 2020). Indeed, biophysical modeling of the different LHb cell types shows that none of them adopt a morphology suited to build-up FPs, since the elementary synaptic dipoles cancel out in cell-size volumes (Figure 8B). Thus, FPs from an alien source colonize the LHb region and are used as if they were generated there *(cuckoo potentials)* (Figure 8C). Spike-phase correlations of LHb cells are therefore intrinsically spurious but may display a correct relationship. However, the risk is that theta activity may continue in the hippocampus whereas the theta phase-lock of Hb cells may end due to modifications to the output of Hb neurons as a result of their synaptic activity, leading to an erroneous interpretation of the theta modulations there. Some of the behavioral or state-dependent modulation described in the literature may reflect this type of phenomenon and needs to be reevaluated, exploring the origin of the FPs.

### 3.8. Population architecture influences waveform parameters: Offset from coherence

We have described how the curved nature of the hippocampus promotes a preferred spread of FPs to structures on the concave side (Figure 6), although other effects related to strong curvatures can be found inside the hippocampus itself. For instance, the folded layer formed by the contiguous CA3 and CA1 populations of pyramidal cells establishes a striking situation in which FPs generated in the former offset others generated in the latter (Martín-Vázquez et al., 2016). For this to happen, the sources in the two regions must be coherent over time, a condition that is fulfilled by CA3-specific (Schaffer) potentials that receive FPs from recurrent excitation of the CA3 pyramidal cells themselves. The result is a layer-dependent change in the amplitude of Schaffer-specific gamma oscillations and sharp-wave potentials in the CA1 area, including a shift of the reversal site of polarity. Note such an effect does not apply to other waves generated by different synaptic inputs to the same cells in the CA1, since they are not coherent (Benito et al., 2014, 2016). These waves of different origin, will suffer a space-dependent waveform change (Martín-Vázquez et al., 2013).

## 4. Concluding remarks

The traditional treatment of FPs is based on an analysis of the temporal fluctuations observed at a specific site. However, their interpretation is affected by several technical and intrinsic difficulties (reviewed in Herreras, 2016). From our point of view, the most ingrained mistake is to treat FPs without taking into account that they are elusive reflections of the real current sources. If FPs were to be generated by a single source, the amplitude would be proportional everywhere, regardless of the geometry, and the spatial rate of decay would not be much of a problem. But they are multi-sourced and the geometry of each one counts to compose a different mix in different recording locations (Herreras et al., 2022). Certainly, current sources cannot be accessed directly with the devices currently available, although spatial information about them and highly accurate time courses can already be obtained through multisite recording and spatial discrimination techniques.

Source geometry takes effect at different levels and affects multiple issues. It is not simply the 3D boundaries encroaching on the zones where active populations inject or drain current (sources and sinks). Indeed, the current density and spatial arrangement of such zones turns out to be critical as it explains the differential decay of FPs in distinct directions. Note that this could be erroneously taken as a manifestation of tissue anisotropy. Also, we should note that the relevant geometry is not that of the active population, since the spatial blocks of current density in the extracellular space result from intense cancellation of microscopic sources and sinks, after which the blocks of positive and negative current may adopt different geometries and densities. Therefore, instead of speaking of favorable or unfavorable structures for the production and spread of FPs in the volume we should rather refer to the specific afferent pathways. The pathway-to-population specific notion of brain sources is an important conceptual asset that facilitates a better understanding of the multiplicity of non-intuitive observations, some of which we discuss here. For example, it helps in the reconceptualization of the so-called LFPs, which, as currently used, do not indicate any of the essential characteristics of the sources, not even their composite nature. By contrast to what is widely believed, the strong potential gradients inside the source boundaries along with the rapid changes in the activation of each of the concurrent pathways makes the single-site recording of potentials in or near the sources a cauldron of uncertainty. Conveniently, the specific geometry of stratified sources is easily implemented in large-scale models that help to understand and unravel phenomena like self-contamination, cuckoo potentials, mutual or unidirectional contamination between brain structures, lateral contamination between cortical zones, the far-reaching spread of potentials from curved structures and others to be described in the future. We think that the spatial dimension of current sources opens a new era that will obviously bring additional complication for study, but will also illuminate brain physiology by providing a solid link between activity of brain circuits and behavior.

## Author contributions

OH and JM were responsible for the concept and drawing the illustrations. OH contributed to information interpretation, editing, and critical revision of the manuscript. All authors revised and approved the final manuscript.

## References

[B1] Abeles M. (1991). *Corticonics: neural circuits of the cerebral cortex.* Cambridge: Cambridge University Press.

[B2] Adey W. R. Kado R. T. McIlwain J. T. Walter D. O. (1966). The role of neuronal elements in regional cerebral impedance changes in alerting, orienting and discriminative responses. *Exp. Neurol.* 15 490–510. 10.1016/0014-4886(66)90146-4 5911102

[B3] Aizawa H. Yanagihara S. Kobayashi M. Niisato K. Takekawa T. Harukuni R. (2013). The synchronous activity of lateral habenular neurons is essential for regulating hippocampal theta oscillation. *J. Neurosci.* 33 8909–8921. 10.1523/JNEUROSCI.4369-12.2013 23678132 PMC6618841

[B4] Anastassiou C. A. Koch C. (2015). Ephaptic coupling to endogenous electric field activity: why bother? *Curr. Opin. Neurobiol.* 31 95–103. 10.1016/j.conb.2014.09.002 25265066

[B5] Arcaro M. J. Livingston M. S. (2021). On the relationship between maps and domains in inferotemporal cortex. *Nat. Rev. Neurosci.* 22 573–583. 10.1038/s41583-021-00490-4 34345018 PMC8865285

[B6] Bédard C. Kröger H. Destexhe A. (2004). Modeling extracellular field potentials and the frequency-filtering properties of extracellular space. *Biophys. J.* 86 1829–1842. 10.1016/S0006-3495(04)74250-2 14990509 PMC1304017

[B7] Bédard C. Piette C. Venance L. Destexhe A. (2022). Extracellular and intracellular components of the impedance of neural tissue. *Biophys. J.* 121 869–885. 10.1016/j.bpj.2022.02.022 35182541 PMC8943819

[B8] Behabadi B. F. Mel B. W. (2014). Mechanisms underlying subunit independence in pyramidal neuron dendrites. *Proc. Natl. Acad. Sci. U.S.A.* 111 498–503. 10.1073/pnas.1217645111 24357611 PMC3890819

[B9] Bell A. Sejnowski T. (1995). An information-maximization approach to blind separation and blind deconvolution. *Neural. Comput.* 7 1129–1159. 10.1162/neco.1995.7.6.1129 7584893

[B10] Benito N. Fernández-Ruiz A. Makarov V. A. Makarova J. Korovaichuk A. Herreras O. (2014). Spatial modules of coherent activity in pathway-specific LFPs in the hippocampus reflect topology and different modes of presynaptic synchronization. *Cerebral Cortex* 24 1738–1752. 10.1093/cercor/bht022 23395845

[B11] Benito N. Martín-Vázquez G. Makarova J. Makarov V. A. Herreras O. (2016). The right hippocampus leads the bilateral integration of gamma-parsed lateralized information. *eLife* 5:e16658. 10.7554/eLife.16658 27599221 PMC5050016

[B12] Berens P. Keliris G. A. Ecker A. S. Logothetis N. K. Tolias A. S. (2008). Comparing the feature selectivity of the gamma-band of the local field potential and the underlying spiking activity in primate visual cortex. *Front. Syst. Neurosci.* 2:2008. 10.3389/neuro.06.002.2008 18958246 PMC2526275

[B13] Bernardi G. Siclari F. Handjaras G. Riedner B. A. Tononi G. (2018). Local and widespread slow waves in stable NREM sleep: evidence for distinct regulation mechanisms. *Front. Hum. Neurosci.* 12:248. 10.3389/fnhum.2018.00248 29970995 PMC6018150

[B14] Bertone-Cueto N. I. Makarova J. Mosqueira A. García-Violini D. Sanchez-Peña R. S. Herreras O. (2020). Volume conducted origin of the field potential at the lateral habenula. *Front. Neurosci.* 13:78. 10.3389/fnsys.2019.00078 31998083 PMC6961596

[B15] Borst J. G. Leung L. W. MacFabe D. F. (1987). Electrical activity of the cingulate cortex. II. cholinergic modulation. *Brain Res.* 407 81–93. 10.1016/0006-8993(87)91221-2 3580858

[B16] Brankaèk J. Stewart M. Fox S. E. (1999). Current source density analysis of the hippocampal theta rhythm: associated sustained potentials and candidate synaptic generators. *Brain Res.* 615 310–327. 10.1016/0006-8993(93)90043-m 8364740

[B17] Buzsáki G. (2002). Theta oscillations in the hippocampus. *Neuron* 33 325–340. 10.1016/s0896-6273(02)00586-x 11832222

[B18] Carmichael J. E. Gmaz J. M. van der Meer M. A. A. (2017). Gamma oscillations in the rat ventral striatum originate in the piriform cortex. *J. Neurosci.* 37 7962–7974. 10.1523/JNEUROSCI.2944-15.2017 28716962 PMC6596914

[B19] Çelik E. Keles U. Kiremitçi I. Gallant J. L. Çukur T. (2021). Cortical networks of dynamic scene category representation in the human brain. *Cortex* 143 127–147. 10.1016/j.cortex.2021.07.008 34411847 PMC8501312

[B20] Chatzikalymniou A. P. Gumus M. Skinner F. K. (2021). Linking minimal and detailed models of CA1 microcircuits reveals how theta rhythms emerge and their frequencies controlled. *Hippocampus* 31 982–1002. 10.1002/hipo.23364 34086375

[B21] Choi S. Cichocki A. Park H. M. Lee S. Y. (2005). Blind source separation and independent component analysis: a review. *Neur. Inf. Proc. Let. Rev.* 6 1–57.

[B22] DeCoteau W. E. Thorn C. Gibson D. J. Courtemanche R. Mitra P. Kubota Y. (2007). Learning-related coordination of striatal and hippocampal theta rhythms during acquisition of a procedural maze task. *Proc. Natl. Acad. Sci. U.S.A.* 104 5644–5649. 10.1073/pnas.0700818104 17372196 PMC1838454

[B23] Destexhe A. Contreras D. Steriade M. (1999). Spatiotemporal analysis of local field potentials and unit discharges in cat cerebral cortex during natural wake and sleep states. *J. Neurosci.* 19 4595–4608. 10.1523/JNEUROSCI.19-11-04595.1999 10341257 PMC6782626

[B24] Elul R. (1962). Dipoles of spontaneous activity in the cerebral cortex. *Exp. Neurol.* 6 285–299.

[B25] Elul R. (1971). The genesis of the EEG. *Int. Rev. Neurobiol.* 15 228–272. 10.1016/s0074-7742(08)60333-5 4949975

[B26] Fein G. Raz J. Brown F. F. Merrin E. L. (1988). Common reference coherence data are confounded by power and phase effects. *Electroenceph. Clin. Neurophysiol.* 69 581–584. 10.1016/0013-4694(88)90171-x 2453336

[B27] Fernández-Ruiz A. Makarov V. A. Benito N. Herreras O. (2012). Schaffer-specific local field potentials reflect discrete excitatory events at gamma-frequency that may fire postsynaptic hippocampal CA1 units. *J. Neurosci.* 32 5165–5176. 10.1523/JNEUROSCI.4499-11.2012 22496562 PMC6622107

[B28] Fernández-Ruiz A. Muñoz S. Sancho M. Makarova J. Makarov V. A. Herreras O. (2013). Cytoarchitectonic and dynamic origins of giant positive LFPs in the dentate gyrus. *J. Neurosci.* 33 15518–15532. 10.1523/jneurosci.0338-13.2013 24068819 PMC6618450

[B29] Florian G. Andrew C. Pfurtscheller G. (1998). Do changes in coherence always reflect changes in functional coupling? *Electroencephalogr. Clin. Neurophysiol.* 106 87–91. 10.1016/s0013-4694(97)00105-3 9680169

[B30] Frostig R. D. Xiong Y. Chen-Bee C. H. Kvasnak E. Stehberg J. (2008). Large-scale organization of rat sensorimotor cortex based on a motif of large activation spreads. *J. Neurosci.* 28 13274–13284. 10.1523/JNEUROSCI.4074-08.2008 19052219 PMC2710304

[B31] Glasgow S. D. Chapman C. A. (2007). Local generation of theta-frequency EEG activity in the parasubiculum. *J. Neurophysiol.* 97 3868–3879. 10.1371/journal.pone.0058901 17392407

[B32] Gómez-Galán M. Makarova J. Llorente-Folch I. Saheki T. Pardo B. Satrústegui J. (2012). Altered postnatal development of cortico-hipocampal neuronal electric activity in mice deficient for the mitochondrial aspartate-glutamate transporter. *J. Cereb. Blood Flow Metab.* 32 306–317. 10.1038/jcbfm.2011.129 21934695 PMC3272597

[B33] Güllmar D. Haueisen J. Reichenbach J. (2015). Influence of anisotropic electrical conductivity in white matter tissue on the EEG/MEG forward and inverse solution. A high-resolution whole head simulation study. *Neuroimage* 51 145–163. 10.1016/j.neuroimage.2010.02.014 20156576

[B34] Haberly L. B. Shepherd G. M. (1973). Current density analysis of summed evoked potentials in opossum prepyriform cortex. *J. Neurophysiol.* 36 789–803. 10.1152/jn.1973.36.4.789 4713320

[B35] Haegens S. Barczak A. Musacchia G. Lipton M. L. Mehta A. D. Lakatos P. (2015). Laminar profile and physiology of the α rhythm in primary visual, auditory, and somatosensory regions of neocortex. *J. Neurosci.* 35 14341–14352. 10.1523/JNEUROSCI.0600-15.2015 26490871 PMC4683691

[B36] Hales C. G. Pockett S. (2014). The relationship between local field potentials (LFPs) and the electromagnetic fields that give rise to them. *Front. Syst. Neurosci.* 8:233. 10.3389/fnsys.2014.00233 25565988 PMC4264498

[B37] Hanson T. L. Fuller A. M. Lebedev M. A. Turner D. A. Nicolelis M. A. (2012). Subcortical neuronal ensembles: an analysis of motor task association, tremor, oscillations, and synchrony in human patients. *J. Neurosci.* 32 8620–8632. 10.1523/JNEUROSCI.0750-12.2012 22723703 PMC3502028

[B38] Herreras O. (2016). Local field potentials: myths and misunderstandings. *Front. Neural. Circ.* 10:101. 10.3389/fncir.2016.00101 28018180 PMC5156830

[B39] Herreras O. Makarova J. Makarov V. A. (2015). New uses for LFPs: pathway-specific threads obtained through spatial discrimination. *Neuroscience* 310 486–503. 10.1016/j.neuroscience.2015.09.054 26415769

[B40] Herreras O. Torres D. Martín-Vázquez G. Hernández-Recio S. López-Madrona V. J. Benito N. (2022). Site-dependent shaping of field potential waveforms. *Cerebral Cortex* 2022:297. 10.1093/cercor/bhac297 35972425 PMC10068269

[B41] Hinman J. R. Penley S. C. Long L. L. Escabí M. A. Chrobak J. J. (2011). Septotemporal variation in dynamics of theta: speed and habituation. *J. Neurophysiol.* 105 2675–2686. 10.1152/jn.00837.2010 21411562

[B42] Hoeltzell P. B. Dykes R. W. (1979). Conductivity in the somatosensory cortex of the cat evidence for cortical anisotropy. *Brain Res.* 177 61–82. 10.1152/jn.1980.43.6.1527 497825

[B43] Holcman D. Yuste R. (2015). The new nanophysiology: regulation of ionic flow in neuronal subcompartments. *Nat. Rev. Neurosci.* 16 685–692. 10.1038/nrn4022 26462753

[B44] Hughes S. W. Lörinc Z. M. Cope D. W. Blethyn K. L. Kékesi K. A. Parri H. R. (2004). Synchronized oscillations at alpha and theta frequencies in the lateral geniculate nucleus. *Neuron* 42 253–268. 10.1016/s0896-6273(04)00191-6 15091341

[B45] Kang D. Ding M. Topchiy I. Shifflett L. Kocsis B. (2015). Theta-rhythmic drive between medial septum and hippocampus in slow-wave sleep and microarousal: a granger causality analysis. *J. Neurophysiol.* 114 2797–2803. 10.1152/jn.00542.2015 26354315 PMC4737409

[B46] Katzner S. Nauhaus I. Benucci A. Bonin V. Ringach D. L. Carandini M. (2009). Local origin of field potentials in visual cortex. *Neuron* 61 35–41. 10.1016/j.neuron.2008.11.016 19146811 PMC2730490

[B47] Korovaichuk A. Makarova J. Makarov V. A. Benito N. Herreras O. (2010). Minor contribution of principal excitatory pathways to hippocampal LFPs in the anesthetized rat: a combined independent component and current source density study. *J. Neurophysiol.* 104 484–497. 10.1152/jn.00297.2010 20463202

[B48] Lalla L. Rueda Orozco P. E. Jurado-Parras M. T. Brovelli A. Robbe D. (2017). Local or not local: investigating the nature of striatal theta oscillations in behaving rats. *eNeuro* 4:ENEURO.0128-17.2017. 10.1523/ENEURO.0128-17.2017 28966971 PMC5616191

[B49] Łȩski S. Lindén H. Tetzlaff T. Pettersen K. H. Einevoll G. T. (2013). Frequency dependence of signal power and spatial reach of the local field potential. *PLoS Comput. Biol.* 9:e1003137. 10.1371/journal.pcbi.1003137 23874180 PMC3715549

[B50] Leung S. W. (1979). Potentials evoked by alvear tract in hippocampal CA1 region of rats. II. *Spat. Field Analy. J. Neurophysiol.* 42 1571–1589. 10.1152/jn.1979.42.6.1571 501390

[B51] Li C. L. Bak A. F. Parker L. O. (1968). Specific resistivity of the cerebral cortex and white matter. *Exp. Neurol.* 20 544–557. 10.1016/0014-4886(68)90108-8 5659447

[B52] Liu X. Zhou L. Ding F. Wang Y. Yan J. (2015). Local field potentials are local events in the mouse auditory cortex. *Eur. J. Neurosci.* 42 2289–2297. 10.1111/ejn.13003 26112462 PMC5014213

[B53] López-Aguado L. Ibarz J. M. Herreras O. (2001). Activity-dependent changes of tissue resistivity in the CA1 region in vivo are layer-specific: modulation of evoked potentials. *Neuroscience* 108 249–262. 10.1016/s0306-4522(01)00417-1 11734358

[B54] López-Aguado L. Ibarz J. M. Varona P. Herreras O. (2002). Structural inhomogeneities differentially modulate action currents and population spikes initiated in the axon or dendrites. *J. Neurophysiol.* 88 2809–2820. 12424314 10.1152/jn.00183.2002

[B55] López-Madrona V. J. Canals S. (2021). Functional interactions between entorhinal cortical pathways modulate theta activity in the hippocampus. *Biology* 10:692. 10.3390/biology10080692 34439925 PMC8389192

[B56] López-Madrona V. J. Pérez-Montoyo E. Álvarez-Salvado E. Moratal D. Herreras O. Pereda E. (2020). Different theta frameworks coexist in the rat hippocampus and are coordinated during memory-guided and novelty tasks. *eLife* 9:e57313. 10.7554/eLife.57313 32687054 PMC7413668

[B57] Lorente de Nó R. (1947). “Analysis of the distribution of action currents of nerves in volume conductors,” in *A study of nerve physiology*, Vol. 132, eds. De Nó R. L. Davis L. (NewYork, NY: The Rockefeller Institute), 384–477. 20261890

[B58] Lubenov E. V. Siapas A. G. (2009). Hippocampal theta oscillations are travelling waves. *Nature* 459 534–539. 10.1038/nature08010 19489117

[B59] Makarov V. A. Makarova J. Herreras O. (2010). Disentanglement of local field potential sources by independent component analysis. *J. Comput. Neurosci.* 29 445–457. 10.1007/s10827-009-0206-y 20094907

[B60] Makarova J. Gómez-Galán M. Herreras O. (2008). Variations in tissue resistivity and in the extension of activated neuron domains shape the voltage signal during spreading depression in the CA1 in vivo. *Eur. J. Neurosci.* 27 444–456. 10.1111/j.1460-9568.2008.06022.x 18215240

[B61] Makarova J. Ibarz J. M. Makarov V. A. Benito N. Herreras O. (2011). Parallel readout of pathway-specific inputs to laminated brain structures. *Front. Syst. Neurosci.* 5:77. 10.3389/fnsys.2011.00077 21949504 PMC3171694

[B62] Makarova J. Ortuño T. Korovaichuk A. Cudeiro J. Makarov V. A. Rivadulla C. (2014). Can pathway-specific LFPs be obtained in cytoarchitectonically complex structures? *Front. Syst. Neurosci.* 8:66. 10.3389/fnsys.2014.00066 24822038 PMC4013467

[B63] Martín-Vázquez G. Asabuki T. Isomura Y. Fukai T. (2018). Learning task-related activities from independent local-field-potential components across motor cortex layers. *Front. Neurosci.* 12:429. 10.3389/fnins.2018.00429 29997474 PMC6028710

[B64] Martín-Vázquez G. Benito N. Makarov V. A. Herreras O. Makarova J. (2016). Diversity of LFPs activated in different target regions by a common CA3 input. *Cerebral Cortex* 26 4082–4100. 10.1093/cercor/bhv211 26400920

[B65] Martín-Vázquez G. Makarova J. Makarov V. A. Herreras O. (2013). Determining the true polarity and amplitude of synaptic currents underlying gamma oscillations of local field potentials. *PLoS One* 8:e75499. 10.1371/journal.pone.0075499 24073269 PMC3779195

[B66] Massimini M. Huber R. Ferrarelli F. Hill S. Tononi G. (2004). The sleep slow oscillation as a traveling wave. *J. Neurosci.* 24 6862–6870. 10.1523/JNEUROSCI.1318-04.2004 15295020 PMC6729597

[B67] McCaan H. Pisano G. Beltrachini L. (2019). Variation in reported human head tissue electrical conductivity values. *Brain Topogr.* 32 825–858. 10.1007/s10548-019-00710-2 31054104 PMC6708046

[B68] Mitzdorf U. (1985). Current source-density method and application in cat cerebral cortex: investigation of evoked potentials and EEG phenomena. *Physiol. Rev.* 65 37–100. 10.1152/physrev.1985.65.1.37 3880898

[B69] Mollazadeh M. Aggarwal V. Davidson A. G. Law A. J. Thakor N. V. Schieber M. H. (2011). Spatiotemporal variation of multiple neurophysiological signals in the primary motor cortex during dexterous reach-to-grasp movements. *J. Neurosci.* 31 15531–15543. 10.1523/JNEUROSCI.2999-11.2011 22031899 PMC3246371

[B70] Montgomery S. M. Betancur M. I. Buzsáki G. (2009). Behavior-dependent coordination of multiple theta dipoles in the hippocampus. *J. Neurosci.* 29 1381–1394. 10.1523/JNEUROSCI.4339-08.2009 19193885 PMC2768079

[B71] Ness T. V. Halnes G. Næss S. Pettersen K. H. Einevoll G. T. (2022). Computing extracellular electric potentials from neuronal simulations. *Adv. Exp. Med. Biol.* 1359 179–199. 10.1007/978-3-030-89439-9_8 35471540

[B72] Nguyen C. V. Müller C. Wolfenstetter T. Yanovsky Y. Draguhn A. Tort A. B. (2016). Hippocampal respiration-driven rhythm distinct from theta oscillations in awake mice. *J. Neurosci.* 36 162–177. 10.1523/JNEUROSCI.2848-15.2016 26740658 PMC6601786

[B73] Nunez P. L. Srinivasan R. (2006). *Electric fields in the brain. the neurophysics of EEG.* Oxford: University Press.

[B74] Okada Y. C. Huang J.-C. H. Rice M. E. Tranchina D. Nicholson C. (1994). Origin of the apparent tissue conductivity in the molecular and granular layers of the in vitro turtle cerebellum and the interpretation of current source-density analysis. *J. Neurophysiol.* 72 742–753. 10.1152/jn.1994.72.2.742 7983532

[B75] Orczyk J. J. Barczak A. Costa-Faidella J. Kajikawa Y. (2021). Cross laminar traveling components of field potentials due to volume conduction of non-traveling neuronal activity in macaque sensory cortices. *J. Neurosci.* 41 7578–7590. 10.1523/JNEUROSCI.3225-20.2021 34321312 PMC8425975

[B76] Ortuño T. López-Madrona V. J. Tapia-González S. Muñoz A. De Felipe J. Herreras O. (2019). Slow-wave activity in the S1HL cortex is contributed by different layer-specific field potential sources during development. *J. Neurosci.* 39 8900–8915. 10.1523/JNEUROSCI.1212-19.2019 31548234 PMC6832678

[B77] Papadimitriou C. H. Vempala S. S. Mitropolsky D. Collins M. Maass W. (2020). Brain computation by assemblies of neurons. *Proc. Natl. Acad. Sci. U.S.A.* 117 14464–14472. 10.1073/pnas.2001893117 32518114 PMC7322080

[B78] Parabucki A. Lampl I. (2017). Volume conduction coupling of whisker-evoked cortical LFP in the mouse olfactory bulb. *Cell Rep.* 21 919–925. 10.1016/j.celrep.2017.09.094 29069599

[B79] Patxinos G. Watson Ch. (2006). *The rat brain in stereotaxic coordinates*. 6th Edn. Amsterdam: Elsevier.

[B80] Pestche H. Stumpf C. (1960). Topographic and toposcopic study of origin and spread of the regular synchronized arousal pattern in the rabbit. *Electroencephalogr. Clin. Neurophysiol.* 12 589–600. 10.1016/0013-4694(60)90101-2 14432410

[B81] Plonsey R. (1964). Volume conductor fields of action currents. *Biophys. J.* 4 317–328. 10.1016/s0006-3495(64)86785-0 14197790 PMC1367509

[B82] Rall W. (1967). Distinguishing theoretical synaptic potentials computed for different soma-dendritic distributions of synaptic input. *J. Neurophysiol.* 30 1138–1168. 10.1152/jn.1967.30.5.1138 6055351

[B83] Rall W. (1977). “Core conductor theory and cable properties of neurons,” in *Handbook of Physiology: The Nervous System*, Vol. 1 ed. Poeter R. (American Physiological Society), 39–97.

[B84] Ranta R. Le Cam S. Tyvaert L. Louis-Dorr V. (2017). Assessing human brain impedance using simultaneous surface and intracerebral recordings. 2017. *Neuroscience* 343 411–422. 10.1016/j.neuroscience.2016.12.013 28012868

[B85] Reimann M. W. Anastassiou C. A. Perin R. Hill S. L. Markram H. Koch C. (2013). A biophysically detailed model of neocortical local field potentials predicts the critical role of active membrane currents. *Neuron* 79 375–390. 10.1016/j.neuron.2013.05.023 23889937 PMC3732581

[B86] Riedner B. A. Hulse B. K. Murphy M. J. Ferrarelli F. Tononi G. (2011). Temporal dynamics of cortical sources underlying spontaneous and peripherally evoked slow waves. *Prog. Brain Res.* 193 201–218. 10.1016/B978-0-444-53839-0.00013-2 21854964 PMC3160723

[B87] Rogers N. Hermiz J. Ganji M. Kaestner E. Kılıç K. Hossain L. (2019). Correlation structure in micro-ECoG recordings is described by spatially coherent components. *PLoS Comput. Biol* 15:e1006769. 10.1371/journal.pcbi.1006769 30742605 PMC6386410

[B88] Rojas-Líbano D. Frederick D. E. Egaña J. I. Kay L. M. (2014). The olfactory bulb theta rhythm follows all frequencies of diaphragmatic respiration in the freely behaving rat. *Front. Behav. Neurosci.* 8:214. 10.3389/fnbeh.2014.00214 24966821 PMC4053074

[B89] Sakurai Y. (1998). The search for cell assemblies in the working brain. *Behav. Brain Res.* 91 1–13. 10.1016/s0166-4328(97)00106-x 9578434

[B90] Sánchez-Vives M. V. Massimini M. Mattia M. (2017). Shaping the default activity pattern of the cortical network. *Neuron* 94 993–1001. 28595056 10.1016/j.neuron.2017.05.015

[B91] Savtchenko L. P. Poo M. M. Rusakov D. A. (2017). Electrodiffusion phenomena in neuroscience: a neglected companion. *Nat. Rev. Neurosci.* 18 598–612. 10.1038/nrn.2017.101 28924257

[B92] Schmidt B. Hinman J. R. Jacobson T. K. Szkudlarek E. Argraves M. Escabí M. A. (2013). Dissociation between dorsal and ventral hippocampal theta oscillations during decision-making. *J. Neurosci.* 33 6212–6224. 10.1523/JNEUROSCI.2915-12.2013 23554502 PMC6618918

[B93] Siapas A. G. Lubenov E. V. Wilson M. A. (2005). Prefrontal phase locking to hippocampal theta oscillations. *Neuron* 46 141–151. 10.1016/j.neuron.2005.02.028 15820700

[B94] Sotero R. C. Bortel A. Naaman S. Mocanu V. M. Kropf P. Villeneuve M. Y. (2015). Laminar distribution of phase-amplitude coupling of spontaneous current sources and sinks. *Front. Neurosci.* 9:454. 10.3389/fnins.2015.00454 26733778 PMC4686797

[B95] Tavares L. C. S. Tort A. B. L. (2022). Hippocampal-prefrontal interactions during spatial decision-making. *Hippocampus* 32 38–54. 10.1002/hipo.23394 34843143

[B96] Toharia P. Robles-Sánchez O. Fernaud-Espinosa I. Makarova J. Galindo S. Rodríguez A. (2016). Pyramidal explorer: a new interactive tool to explore morpho-functional relations of pyramidal neurons. *Front. Neuroanat* 9:159. 10.3389/fnana.2015.00159 26778972 PMC4701943

[B97] Torres D. Makarova J. Ortuño T. Benito N. Makarov V. A. Herreras O. (2019). Local and volume-conducted contributions to cortical field potentials. *Cerebral Cortex* 29 5234–5254. 10.1093/cercor/bhz061 30941394

[B98] Tort A. B. L. Ponsel S. Jessberger J. Yanovsky Y. Brankaèk J. Draguhn A. (2018). Parallel detection of theta and respiration-coupled oscillations throughout the mouse brain. *Sci. Rep.* 8:6432. 10.1038/s41598-018-24629-z 29691421 PMC5915406

[B99] van Daal R. J. J. Sun J. J. Ceyssens F. Michon F. Kraft M. Puers R. (2020). System for recording from multiple flexible polyimide neural probes in freely behaving animals. *J. Neural Eng.* 17 016046. 10.1088/1741-2552/ab5e19 31791021

[B100] Varona P. Ibarz J. M. López-Aguado L. Herreras O. (2000). Macroscopic and subcellular factors shaping CA1 population spikes. *J. Neurophysiol.* 83 2192–2208. 10758128 10.1152/jn.2000.83.4.2192

[B101] Volgushev M. Chauvette S. Timofeev I. (2011). Long-range correlation of the membrane potential in neocortical neurons during slow oscillation. *Prog. Brain Res.* 193 181–199. 10.1016/B978-0-444-53839-0.00012-0 21854963 PMC3397925

[B102] Whitmore N. W. Lin S. C. (2016). Unmasking local activity within local field potentials (LFPs) by removing distal electrical signals using independent component analysis. *Neuroimage* 132 79–92. 10.1016/j.neuroimage.2016.02.032 26899209 PMC4885644

[B103] Woodbury J. W. (1960). “Potentials in a volume conductor,” in *Medical physiology and biophysics*, eds. Ruch T. C. Fulton J. F. (Philadelphia and London: WB Saunders Co), 83–91.

[B104] Xing D. Yeh C. I. Shapley R. M. (2009). Spatial spread of the local field potential and its laminar variation in visual cortex. *J. Neurosci.* 29 11540–11549. 10.1523/JNEUROSCI.2573-09.2009 19759301 PMC2910581

