# Supplementary material for: Theoretical considerations and supporting evidence for the primary role of source geometry on field potential amplitude and spatial extent
Source: Front Cell Neurosci. 2023 Mar 30;17:1129097. doi: 10.3389/fncel.2023.1129097 (PMC10097999; doi:10.3389/fncel.2023.1129097)
Supplement: Supplementary file 1 [file Presentation_1.pdf]

Supplementary Material

Theoretical Considerations and Supporting Evidence for the Primary Role of Source Geometry on Field Potential Amplitude and Spatial Extent

Oscar Herreras,*, Daniel Torres, Valeriy A Makarov, Julia Makarova*

Correspondence*: herreras@cajal.csic.es; Julia.samuseva@cajal.csic.es

1 Supplementary Video 1

Video 1. Three-dimensional representation of the running potentials elicited by cortical and hippocampal neuronal sources of current. The structure of the sources is shown in the background in black and the concentric spheroids represent isopotential surfaces (blue and red are negative and positive values, respectively). The activities modeled are: (right) alfa-gamma motifs in the dentate gyrus; and (left) co-activation of these with slow cortical waves and hippocampal theta rhythm. Traces below correspond to customary single-site recordings in sites indicated above by colored dots. These are from a point in the thalamus (blue) and another in the cortex layer V (red). See Figure 1 in main text for further details. The model is based on finite-element methods (FEMs) using realistic dimensions, current densities and temporal dynamics (Model as in Torres et al., Cerebral Cortex, 29:5234, 2019).

2 Supplementary Video 2

Video 2. Three-dimensional representation of the running potentials elicited by theta currents in the CA1 hippocampal field. The structure of the sources is shown in the background (black) and the concentric spheroids represent isopotential surfaces (blue and red are negative and positive values, respectively). All plots represent hippocampal theta potentials (AC-filtered) elicited by dipolar currents with active inputs in the CA1 st. lacunosum-moleculare over different septotemporal segments: (upper left) dorsal CA1; (upper right), lateral CA1 (vertical limb); (bottom left) dorsal plus lateral; (bottom right) complete CA1 activation. See Figure 7 in main text for further details. The

model is based on finite-element methods (FEMs) using realistic dimensions, current densities and temporal dynamics (Model as in Torres et al., Cerebral Cortex, 29:5234, 2019).

frontiers
